# Indirect Time-of-Flight Depth Sensor with Two-Step Comparison Scheme for Depth Frame Difference Detection

**DOI:** 10.3390/s19173674

**Published:** 2019-08-23

**Authors:** Donguk Kim, Jaehyuk Choi

**Affiliations:** College of Information and Communication Engineering, Sungkyunkwan University, Suwon 16419, Korea

**Keywords:** time-of-flight (TOF), depth sensor, frame difference, CMOS image sensor

## Abstract

A depth sensor with integrated frame difference detection is proposed. Instead of frame difference detection using light intensity, which is vulnerable to ambient light, the difference in depth between successive frames can be acquired. Because the conventional time-of-flight depth sensor requires two frames of depth-image acquisition with four-phase modulation, it has large power consumption, as well as a large area for external frame memories. Therefore, we propose a simple two-step comparison scheme for generating the depth frame difference in a single frame. With the proposed scheme, only a single frame is needed to obtain the frame difference, with less than half of the power consumption of the conventional depth sensor. Because the frame difference is simply generated by column-parallel circuits, no access of the external frame memory is involved, nor is a digital signal processor. In addition, we used an over-pixel metal–insulator–metal capacitor to store temporary signals for enhancing the area efficiency. A prototype chip was fabricated using a 90 nm backside illumination complementary metal–oxide–semiconductor (CMOS) image sensor process. We measured the depth frame difference in the range of 1–2.5 m. With a 10 MHz modulation frequency, a depth frame difference of >10 cm was successfully detected even for objects with different reflectivity. The maximum relative error from the difference of the reflectivity (white and wooden targets) was <3%.

## 1. Introduction

In both three-dimensional (3D) and conventional two-dimensional (2D) imaging, acquiring digital image signals with full spatial resolution is redundant, particularly when the image is utilized only for the recognition of objects and the activation of functions. Instead, the frame difference can be acquired for the recognition and tracking of moving objects, as well as for motion-triggered awakening [1,2,3]. Specifically, acquiring the frame difference suppresses the transmission of redundant information through the identification of moving objects and the elimination of repetitive frames in surveillance systems [4]. In machine vision, successive functions such as the tracking of moving objects can be activated by the frame difference [5]. Another application of the frame difference is the optic-flow sensor for the navigation of micro-vehicles [6]. The on-chip optic-flow generation firstly requires the detection of the frame difference in order to provide the pattern of motion of objects.

For 2D imaging, several image sensors with integrated frame difference detection were reported [1,2,3,4,5,6]. The sensors generate the frame difference by simply subtracting signals of successive frames. However, the frame difference is determined by simply calculating the change in light intensity, which varies according to the ambient light. Thus, the absolute difference for moving objects cannot be acquired. A more critical problem is that the frame difference cannot be detected under dark conditions. Additionally, the optic-flow sensor reported in Reference [6] generates only a 2D optic flow based on the frame difference of the light intensity, which is inaccurate when the ambient light intensity is extremely low or extremely high in outdoor applications.

Three-dimensional imaging that provides depth information, as well as 2D shape information, can be implemented with a variety of methods such as structured light projection (SLP), direct time-of-flight (dTOF), and indirect time-of-flight (iTOF) methods. These 3D imaging methods have the advantage of being immune to ambient light because they involve the detection of infrared (IR) light. Moreover, the 3D movement of objects can be detected. The SLP method provides high depth accuracy but involves complex post-processing in order to calculate the depth from the pattern matching [7]. Even though the dTOF method offers simple post processing, it requires photodetectors with high sensitivity (such as avalanche photodiodes and single-photon avalanche diodes) and a large form factor in order to measure the time-of-flight with a small number of incident photons in a single measurement [8,9]. Therefore, high spatial resolution is difficult to be implemented. Among the 3D imaging methods, the iTOF method provides high depth accuracy, simple post processing, and high spatial resolution using small photodetectors (such as pinned photodiodes or photogates) that are widely used in 2D image sensors [10,11,12,13]. In the iTOF depth sensor, the four-phase modulation scheme is usually used to provide an accurate depth regardless of the reflectivity of objects while suppressing the offset from ambient lights. However, this four-phase modulation scheme requires two frames of modulation for acquisition of a single-frame depth image, power consuming modulation and analog-to-digital (A/D) conversion in two frames, and huge frame memory to store intermediate signals, which is illustrated in Section 2.

In this paper, we propose a two-step comparison scheme for detecting an accurate depth frame difference regardless of the reflectivity. Without power-consuming four-phase modulation, A/D conversion, digital readout, and image signal processing, a depth frame difference can be simply generated via two-phase modulation with only column-parallel circuits in a single frame. Moreover, instead of frame memory, we implemented an over-pixel metal–insulator–metal (MIM) capacitor to store previous frame signals. Owing to the backside illumination (BSI) complementary metal–oxide–semiconductor (CMOS) image sensor (CIS) process [3,14], the over-pixel MIM capacitor as an analog memory (AM) did not reduce the sensitivity. Additionally, we reused the existing column-parallel amplifier circuit for the gain amplification of signals and reused the comparator of the column-parallel A/D converter (ADC) for acquiring the depth frame difference without a significant area overhead. 

The remainder of this paper is organized as follows: Section 2 introduces conventional four-phase modulation scheme. Section 3 describes the operation principle of the proposed two-step comparison scheme for acquiring the depth frame difference. Section 4 describes the structure and operation of the circuit. Section 5 presents the experimental results. The paper is concluded in Section 6.

## 2. Conventional Four-Phase Modulation Scheme 

**Figure 1:**
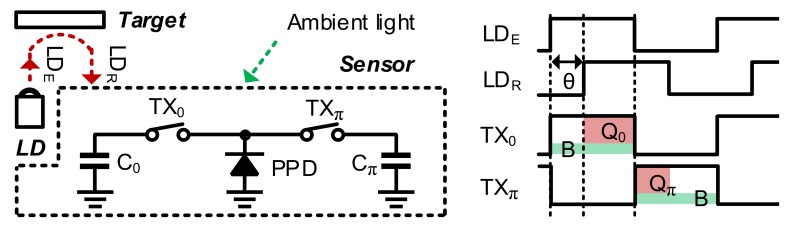
Operation principles of an indirect time-of-flight (iTOF) depth sensor.

As shown in Figure 1, an IR laser diode (LD) emits modulated light. The iTOF sensor calculates the depth according to the phase difference *θ* between the emitted light LD_E_ and reflected light LD_R_. Two electronic shutters TX_0_ and TX_π_ in a pixel are modulated in-phase (synchronized with the LD) and out-of-phase, respectively. The typical modulation frequency is >10 MHz. The sensor detects a photogenerated current (I_PIX_) in a pinned photodiode (PPD), which is integrated to obtain the charges *Q*_0_ from TX_0_ and *Q_π_* from TX_π_. Then, *θ* can be calculated as follows:

(1) $$\theta =\pi \cdot \frac{AQ_{\pi }}{AQ_{0}+AQ_{\pi }}=\frac{Q_{\pi }}{Q_{0}+Q_{\pi }}=\pi \cdot \frac{Q_{\pi }}{Q_{TOT}},$$

where *A* represents the gain from different reflectivities and distances of the object. The total charge *Q_TOT_* that is the sum of *Q*_0_ and *Q_π_* varies according to the distance and the reflectivity. However, the ratio *Q_π_*/*Q_TOT_* depends only on the distance. This operation is called two-phase modulation. Unfortunately, there is a strong background signal from ambient light, particularly in outdoor applications. This strong ambient light provides a common direct-current (DC) offset (*BG*) to *Q*_0_ and *Q_π_*, as shown in Figure 1. Accordingly, *θ* becomes erroneous, as shown in Equation (2).

(2) $$\theta +error=\pi \cdot \frac{AQ_{\pi }+BG}{AQ_{0}+BG+AQ_{\pi }+BG}=\pi \cdot \frac{AQ_{\pi }+BG}{AQ_{TOT}+2BG}.$$

Therefore, in the conventional iTOF sensor, four-phase modulation is commonly used. To cancel the background signal *BG*, Δ*Q*_0_ = *AQ*_0_ − *AQ_π_* is acquired. To cancel the gain term *A*, we obtain another signal Δ*Q_π_*_/2_ = *AQ*_*π*/2_ − *AQ*_3*π*/2_ in the next frame. Finally, we can obtain the exact distance regardless of the reflectivity and ambient light [13] by calculating the following ratio:

(3) $$\theta =\frac{\pi }{2}\times \left(1-\frac{\Delta Q_{\pi /2}}{\left|\Delta Q_{0}\right|+\left|\Delta Q_{\pi /2}\right|}\right).$$

To calculate the depth frame difference, we must calculate and store *θ*_(1)_ in Frame #1, calculate *θ*_(2)_ in Frame #2, and then detect their difference. However, we have three critical problems. Firstly, we need two frames to obtain *θ*_(1)_ (and also *θ*_(2)_) for the four-phase modulation. This two-frame operation (for each *θ*_(k)_) requires a large power consumption, particularly for the modulation of pixels. Additionally, the calculation of *θ* involves a digital readout of 10-bit signals and image signal processing, including division, which requires additional power consumption. Secondly, significant motion blur arises because of the two-frame operation, particularly for fast-moving objects. Thirdly, a frame memory with large area is needed to store the Δ*Q*_0_ and Δ*Q_π_*_/2_ generated from the previous frame. In Section 3, we illustrate the proposed two-step comparison scheme that generates the depth frame difference in a single frame without area overhead from the frame memories and power consumption overhead from the modulation in two frames. 

## 3. Two-Step Comparison Scheme for Acquiring Depth Frame Difference

The main purpose of the proposed scheme is to provide on-chip depth frame difference regardless of reflectivity of objects. In ideal case, we can detect the depth frame difference by measuring only intensity of the reflected light from a single object because the light intensity decreases according to the distance. However, more than two objects with different reflectivities provide different intensity (according to the reflectivity). Therefore, a simple measurement of light intensity like a conventional proximity sensor in mobile devices will induce an error for calculating the absolute difference of depth in successive frames. Instead of light intensity, we can calculate the depth itself using the four-phase modulation scheme that is commonly used in an iTOF depth sensor. However, as mentioned in Section 2, significant overhead of area, power consumption, and speed arise. Therefore, we propose the two-step comparison scheme to generate on-chip depth frame difference in a single frame without any additional memory and power consumption overhead from the modulation. 

**Figure 2:**
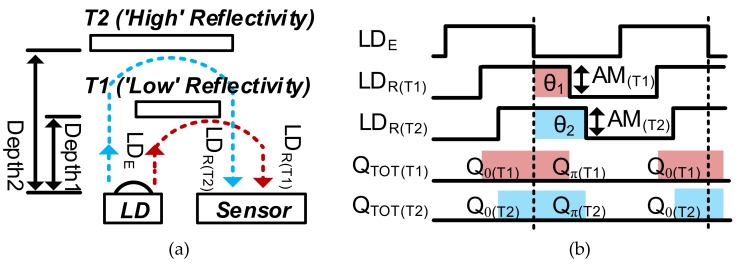

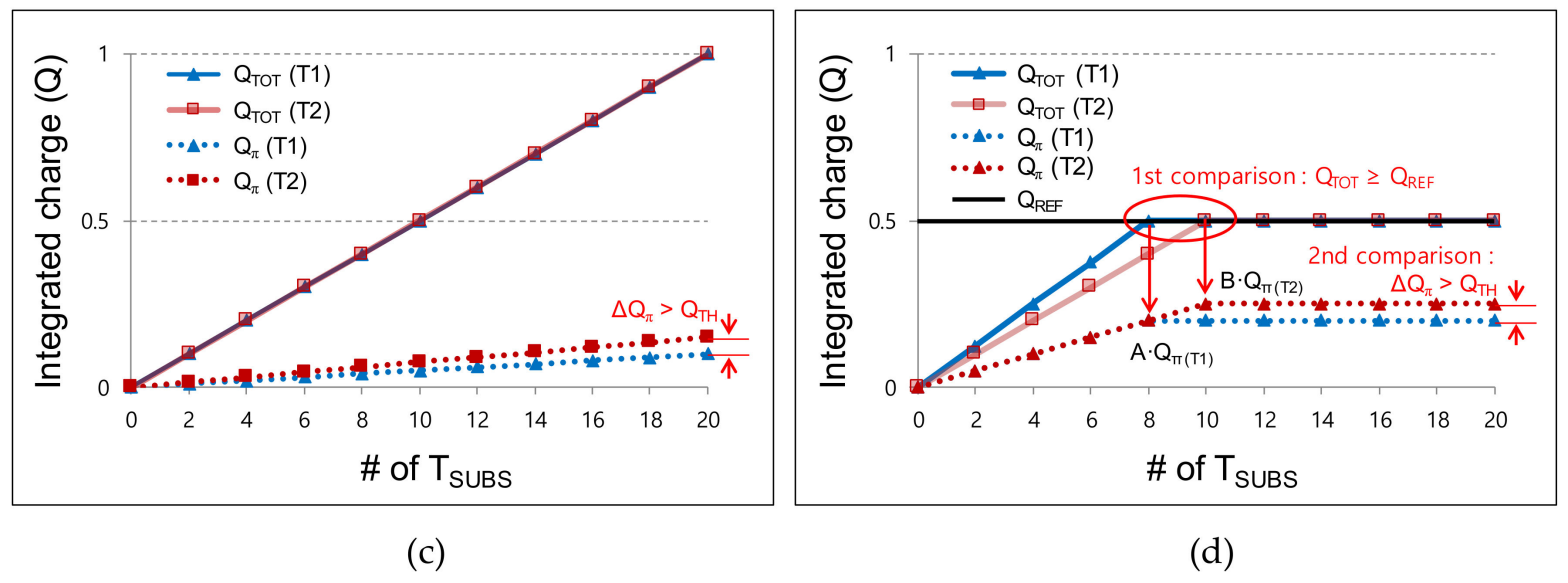
Operation principles for detecting the depth frame difference: (**a**) example of detecting two objects with different reflectivities at different distances; (**b**) timing diagram; (**c**) integrated charges from two objects in the case that provides the same total charges; (**d**) integrated charges from two objects in the case that provides different total charges. For illustration, we described *Q* in the range of 0–1. *Q_π_* is the charge integrated by the out-of-phase (180°) modulation, *Q_TOT_* is the sum of *Q*_0_ and *Q_π_*, and *Q_REF_* is the reference of the first comparison.

Figure 2 shows the operation principles of the proposed method for acquiring the depth frame difference. The main idea is that the change of *Q_π_*/*Q_TOT_* in successive frames is detected if the depth frame difference occurs. Because *Q_TOT_* varies according to the reflectivity, as well as the distance, *Q_TOT_* (and also *Q_π_*) is linearly adjusted to the fixed reference first. Then, the depth frame difference can be detected by simply detecting the change in *Q_π_* because the denominator *Q_TOT_* was adjusted to the fixed reference. For illustration, we assumed a special case in which Targets 1 (T1) and 2 (T2) are present in the first and second frames, respectively, as shown in Figure 2a. The targets have different reflectivities and depths. We assumed this special case to show that the proposed scheme works regardless of the reflectivity of the objects. Assuming that the amplitude (AM_(*T*1)_) of reflected light (LDR_(*T*1)_) from T1 and the amplitude (AM_(*T*2)_) of reflected light LDR_(*T*2)_ from T2 are equal, the total integrated charges *Q_TOT_*_(*T*1)_ and *Q_TOT_*_(*T*2)_ are the same, as shown in Figure 2c. In the illustration of Figure 2c, we describe the integrated charge at each sub-integration time *T_SUB_*, where *T_SUB_* is evenly divided over the whole integration time. The situation illustrated in Figure 2c can occur even with different distances, because of differences in reflectivity. In this case, we cannot detect the depth difference by just calculating the change in the light intensity (*Q_TOT_*) even though *Q_TOT_* varies according to the distance, because the *Q_TOT_* values in successive frames are equal owing to reflectivity. However, using the proposed two-step comparison scheme, we can detect the depth difference for T1 and T2 without depth calculation in Equation (1) regardless of the *Q_TOT_* values. Assuming that *Q_TOT_*_(*T*1)_ and *Q_TOT_*_(*T*2)_ are different, as shown in case 2 of Figure 2d, we equalize *Q_TOT_*_(*T*1)_ and *Q_TOT_*_(*T*2)_ using two phase operations. In the first phase, we integrate charges until *Q_TOT_* reaches the fixed reference *Q_REF_* for equalizing *Q_TOT_*_(*T*1)_ and *Q_TOT_*_(2)_. For this equalization process, the total integration time is divided into *N* sub-integration times (*N*·*T_SUB_*). Each *T_SUB_* consists of the modulation time (*T_MOD_*) for accumulating photogenerated charge in the pixel and the accumulation time for accumulating the pixel output into the AM, as illustrated in detail in Section 4. Therefore, the total charges integrated in the pixel (*Q_TOT_*(*N*·*T_SUB_*)) can be expressed as follows:

(4) $$Q_{TOT}\left(N\cdot T_{SUB}\right)=\overset{N}{\underset{T_{SUB}=0}{\sum }}I_{PIX}\cdot T_{MOD},$$

where *I_PIX_* represents the photocurrent in a pixel. At each *T_SUB_*, *Q_TOT_* is compared with *Q_REF_*. The integration of *Q_TOT_* is continued until the *k-*th *T_SUB_* (*k*·*T_SUB_*) that has a larger *Q_TOT_* than *Q_REF_* is reached. We can then obtain

(5) $$A\cdot Q_{TOT\left(T1\right)}\;=\;B\cdot Q_{TOT\left(T2\right)}\;=\;Q_{REF},$$

where *A* and *B* are proportionality factors (PFs) based on the controlled integration time. This equalization process involves comparison in each *T_SUB_*. We call this process the first comparison phase. In the second comparison phase, the values of *A*·*Q_π_*_(*T*1)_ and *B*·*Q_π_*_(*T*2)_ scaled with same PF used in the first comparison phase are compared. They have hte same PF because *Q_π_* experiences the same controlled integration time (*k*·*T_SUB_*) as *Q_TOT_*. According to the adjusted integration in the first comparison phase, we already have *A*·*Q_π_*_(*T*1)_ and *B*·*Q_π_*_(*T*2)_. Because the denominator *Q_TOT_* in Equation (1) is a constant in the first comparison phase, a simple comparison of the two *Q_π_* values provides the same result as a comparison of the *θ* values. Therefore, we can effectively compare the ratios *Q_π_*_(*T*1)_/*Q_TOT_*_(*T*1)_ and *Q_π_*_(*T*2)_/*Q_TOT_*_(*T*2)_, where the reflectivity in both the numerator and denominator can be divided and cancelled. The *Q_π_*_(1)_ and *Q_π_*_(2)_ from Frames #1 and #2, respectively, are simply compared to determine whether a significant frame difference of the depth occurs, as follows:

(6) $$\left|A\cdot Q_{\pi \left(1\right)}-B\cdot Q_{\pi \left(2\right)}\right|>Q_{th}\;\left(\mathrm{second}\;\mathrm{comparison}\right),$$

where *Q_th_* is the threshold of the depth frame difference. Using this two-step comparison scheme, we can simply generate the depth frame difference without accessing the A/D-converted digital signal and calculating the ratio in the image signal processor. More significantly, the two-phase modulation is sufficient; the power-consuming (and slow) four-phase modulation is not necessary.

**Figure 3:**
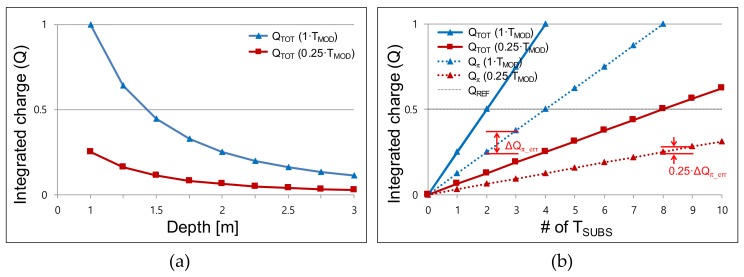
Requirement of the adaptive modulation time with multiple *T_SUB_*s: (**a**) integrated total charge (*Q_TOT_*) according to the distance with the adaptive modulation time; (**b**) integrated *Q_TOT_* and *Q_π_* according to the number of *T_SUB_*s with the adaptive modulation time.

However, the proposed two-step comparison scheme can induce an error because the integrated charge decreases significantly along the distance, particularly for a short distance, as shown in Figure 3a. To illustrate this error, in Figure 3b, we assume that the *Q_TOT_*_(1)_ (marked with a blue line) of the first frame reaches *Q_REF_* (= 0.5) at the second *T_SUB_* and that the *Q_TOT_*_(2)_ of the second frame is exactly equal to *Q_TOT_*_(1)_. Additionally, we assume that the *Q_π_* (marked with a blue dotted line) in both frames is half of the *Q_TOT_*. In this case, no frame difference should be detected. However, if *Q_TOT_*_(2)_ is judged as a smaller value than *Q_REF_* in the second *T_SUB_* owing to noise, *Q_TOT_*_(2)_ is decided as 0.75 in the third *T_SUB_*. In this case, *Q_π_*_(2)_ is 0.375 owing to the error, whereas *Q_π_*_(1)_ is 0.25. With this error (Δ*Q_π_err_* = 0.125), if *Q_TH_* is set as 0.1, the wrong frame difference (0.375 − 0.25 > 0.1) is detected, even though no frame difference occurs. In summary, *Q_TOT_* can be integrated with one more *T_SUB_* via random noise or quantization noise during the first comparison phase. This induces significant error, particularly at a short distance, because *Q* decreases significantly with an increase in the distance, as shown in Figure 3b. 

To suppress this error, we must reduce the increment of *Q* in each *T_SUB_* only for the short distance that induces significant error. This can be achieved by using the adaptive modulation time AF·*T_MOD_*, where AF is an adaptive factor. Therefore, the decrement of *Q* is reduced at a short distance, whereas the decrement of *Q* is maintained (or increased) at a long distance. This effect is also shown in Figure 3a,b. The increment of *Q* (marked with a solid red line for *Q_TOT_* and a dotted red line for *Q_π_*) in each *T_SUB_* is reduced by applying AF·*T_MOD_*/*N* in each *T_SUB_*, where AF < 1. Then, *Q_π_*_(2)_ is 0.28125 in the ninth *T_SUB_* (owing to the error), and *Q_π_*_(1)_ (0.25·*T_MOD_*) is 0.25 in the eighth *T_SUB_*. The depth frame difference (0.28125 − 0.25 < 0.1) is not detected, as desired.

**Figure 4:**
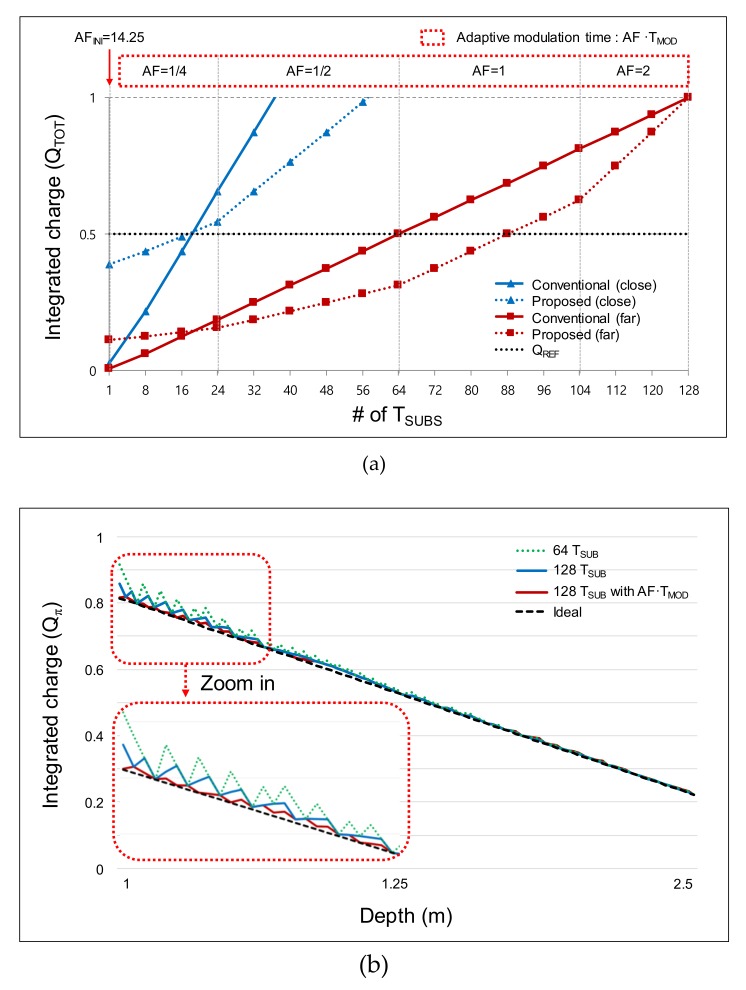
Δ*Q_π_* simulated using the two-step comparison scheme: (**a**) accumulated Δ*Q_π_* according to the accumulation in each sub-integration time *T_SUB_*; (**b**) Δ*Q_π_* acquired from the first comparison phase.

Figure 4a shows the integrated *Q_TOT_* along the sub-integration time. Both the cases with and without the adaptive modulation time are shown. We reduce the increment of *Q* for the first 24 *T_SUB_*s by applying a small AF (*T_MOD_*/4), because the integrated *Q_TOT_* generated from the short-distance objects has an abrupt transition along the distance. In this case, we have a large error that arises from the difference between the integrated *Q_TOT_* (at the 24th *T_SUB_*) and the reference *Q_REF_* (*Q_TOT_* − *Q_REF_*). By reducing the *T_MOD_* to *T_MOD_*/4, the error can be suppressed owing to the decreased increment of *Q*. However, for the long-distance objects, the rate of the charge integration is too low because of the reduced *T_MOD_*. Therefore, from the 25th *T_SUB_* to the 128th *T_SUB_*, we gradually increase the *T_MOD_* from *T_MOD_*/2 to 2·*T_MOD_* such that the integration of the small *Q* reaches *Q_REF_*. Note that only the first *T_SUB_* has a large AF (14.25). This is because the large initial *Q* (integrated in the 1st *T_SUB_*) accelerates the time to reach *Q_REF_* within 128·*T_SUB_*. Without a large AF in the first *T_SUB_*, the integration of the small *Q* (in the case of long-distance objects) does not reach *Q_REF_*.

Figure 4b illustrates the PF·Δ*Q_π_* according to the distance. Using the proposed two-step comparison scheme, we acquire PF·*Q_TOT_* first (first comparison) from *N*·*T_SUB_*. The maximum *Q_TOT_* is set as 1 for simple illustration, as shown in Figure 4a. As shown in Figure 3a, *Q_TOT_* decreases proportionally to the square of the distance. Then, the PF·*Q_TOT_* and the resultant PF·Δ*Q_π_* are acquired according to the distance. We use Δ*Q_π_* (= *Q*_0_ + *BG* − *Q_π_* − *BG* = *Q*_0_ − *Q_π_*) instead of *Q_π_* in this calculation because we actually use Δ*Q_π_* in the prototype chip in order to cancel out the background term *BG*, as in the four-phase modulation. As illustrated in Figure 4b, allocating a larger number of *T_SUB_*s in a given frame suppresses the depth error because the *T_SUB_* is the effective resolution that determines Δ*Q_π_*. With the allocation of 128·*T_SUB_*, the deviation in each *T_SUB_* is suppressed. Without the adaptive modulation time, the error at the short distance (<1.5 m) is large (5.5% at maximum) because of the large decrement of *Q_TOT_*. Using the adaptive modulation time shown in Figure 4a, the large decrement can be suppressed, and the error rate is reduced to 2.2% (maximum) over all distances. Thus, we can detect a frame difference larger than a certain threshold regardless of the distance of the objects. In this way, we suppress the nonlinearity-induced error using the adaptive modulation time and the allocation of 128·*T_SUB_*.

In summary, the depth frame difference can be calculated in a single frame using the proposed two-step comparison scheme without generating four-phase images in two frames. Even though finite error in the depth calculation occurs owing to the quantization, the error can be suppressed below 2.2 % (<3.3 cm) by aid of the adaptive modulation. Because of the single-frame operation, no additional memory and power consumption overhead for the four-phase modulation are required. The detailed circuit implementation of the two-step comparison scheme is illustrated in the next section. 

## 4. Circuit Implementation

**Figure 5:**
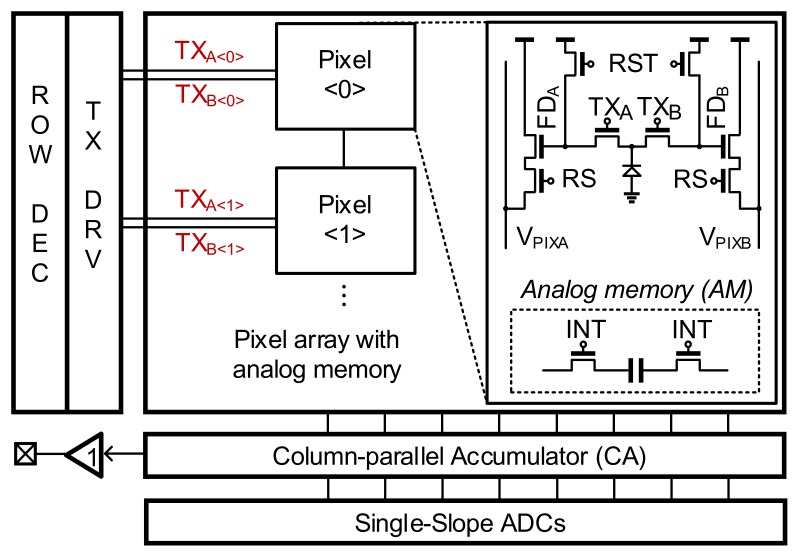
Overall architecture of the sensor.

Figure 5 shows the overall architecture of the proposed sensor chip. The sensor chip consists of an array of pixels with an over-pixel AM, a TX driver, and a row decoder for driving and selecting pixels, a column-parallel accumulator (CA) for accumulating charges from the pixels into the AM, and a unity-gain buffer for the output. The pixel consists of a PPD, two reset transistors (RST), two row-selection transistors (RS), two source follower transistors, and two electronic shutters (TX_0_ and TX_π_). Additionally, the AM (including one capacitor and two access transistors) is placed to store an intermediate *Q* during the integration time. After the overall operation is finished in a frame, this AM stores the acquired Δ*Q_π_* in a given frame, which becomes the previous frame signal in the next frame.

The proposed two-step comparison scheme requires comparing the values of *Q_TOT_* and *Q_π_*. However, with strong ambient light, the DC offset *BG* is added, as indicated by Equation (2). Therefore, instead of acquiring *Q_TOT_* and *Q_π_*, we must obtain Δ*Q_TOT_* = (*Q_TOT_* + *BG*) − (0 + *BG*) and Δ*Q_π_* = (*Q*_0_ + *BG*) − (*Q_π_* + *BG*) in a single frame. Both *Q_π_* and Δ*Q_π_* provide phase information according to the depth [2]. Therefore, the first comparison is performed as Δ*Q_TOT_* > Δ*Q_REF_*, and the second comparison is performed as |*A*·Δ*Q_π_*_(1)_ − *B*·Δ*Q_π_*_(2)_| > Δ*Q_TH_*. 

**Figure 6:**
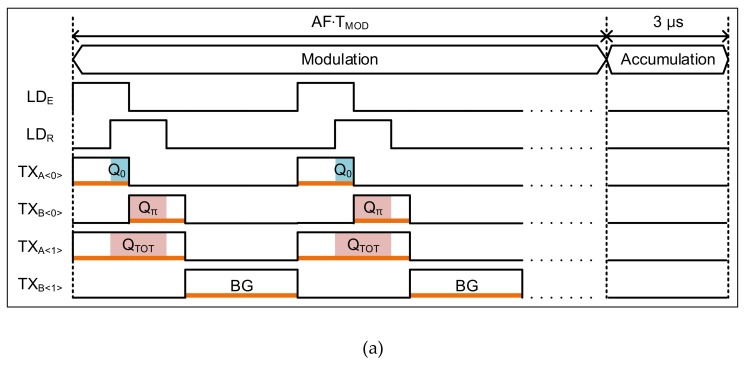

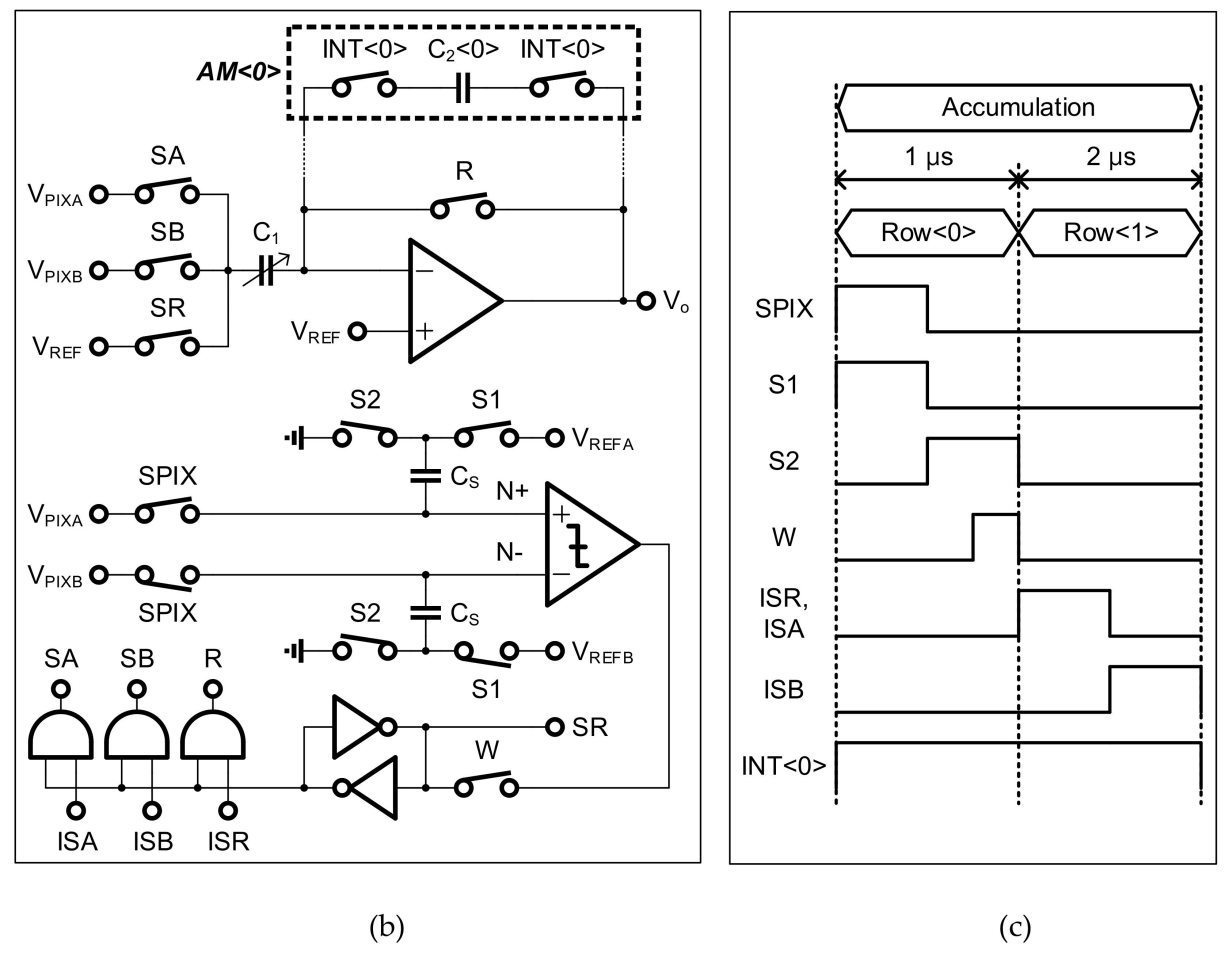
The proposed column-parallel accumulator (CA) circuit and the operation in one *T_SUB_*: (**a**) timing diagram of one *T_SUB_*; (**b**) circuit schematic; (**c**) timing diagram in one accumulation time.

For a two-step comparison scheme, we grouped two adjacent pixels. The pixel<0> generates Δ*Q_TOT_*, and the pixel<1> generates Δ*Q_π_*. As shown in Figure 6a, the modulation period of TX in pixel<1> is twice that of the even pixels, such that Δ*Q_TOT_* is generated in a single frame. Each pixel has an AM, i.e., AM<0> to AM<1>. The Δ*Q_π_*_(1)_s from Frame #1 are stored in AM<0>, and the Δ*Q_π_*_(2)_s from Frame #2 are stored in AM<1>.

Figure 6b shows the CA circuit that accumulates the pixel output into the AM and reads the stored signal in the AM. The CA consists of an analog multiplexer, an amplifier, a static random-access memory (SRAM), and an input capacitor bank (C_1_) for providing a high gain of >8. The comparator circuit that is originally used for the single-slope ADC is reused for the two-step comparison scheme. Additionally, the amplifier originally used for the column-parallel programmable-gain amplifier is reused for area efficiency.

The timing diagram of Figure 6a illustrates the operation in a single *T_SUB_* in one frame. Each *T_SUB_* consists of two operation phases: (1) modulation, and (2) accumulation and the first comparison. The detailed operation is as follows: the first phase is the modulation phase, where *Q*_0_, *Q_π_*, and *Q_TOT_* are integrated in the floating-diffusion (FD) nodes of pixels by modulating the electronic shutters TX_A_ and TX_B_. After the modulation phase, four signals (*Q*_0_ + *BG*, *Q_π_* + *BG*, *Q_TOT_* + *BG*, *BG*) are generated. These signals are stored in the FD nodes FD_A_ and FD_B_ of the two pixels. The second phase is the accumulation and first comparison phase. In the second phase, the integrated Δ*Q_TOT_* (from pixel<0>) until the current *T_SUB_* and Δ*Q_REF_* are compared. If Δ*Q_TOT_* is larger than Δ*Q_REF_*, Δ*Q_π_* (from pixel<1>) is stored in the AM. In this case, Δ*Q_π_* is no longer stored in the AM from the next *T_SUB_*. Therefore, the Δ*Q_π_* acquired when Δ*Q_TOT_* reaches Δ*Q_REF_* is preserved in the AM. When Δ*Q_TOT_* is smaller than Δ*Q_REF_*, Δ*Q_π_* is still stored in the AM. However, in this case, a new Δ*Q_π_* is stored in the AM in the next *T_SUB_*. After 128∙*T_SUB_* (= 1 frame), the Δ*Q_π_* stored in the current frame and the Δ*Q_π_* stored in the previous frame are accessed through the amplifier to be compared. 

The circuit operation, along with a timing diagram, is shown in Figure 6c. At *t*_1_, even pixels (row<0>) are selected. The switches SA and SAD are enabled. Then, two outputs of the source followers (V_PIXA_ and V_PIXB_) are sampled on *C_S_*. At this time, *V_PIXA_* is *V_RST_* − *BG*/*C_FD_*, and *V_PIXB_* is *V_RST_* − (*Q_TOT_* + *BG*)/*C_FD_*. At *t*_2_, the switch SB is enabled. Then, the comparator inputs N+ and N− experience voltage drops based on *V_REFA_* and *V_REFB_*. We set *V_REFB_* as *V_REFA_* + *Q_REF_*/*C_FD_*, such that the comparator compares *Q_TOT_* with *Q_REF_*. Therefore, if *Q_TOT_* > *Q_REF_*, the comparator generates an output of “1”. At *t*_3_, the switch SE is enabled to store the comparator output in the SRAM. At *t*_4_, odd pixels (row<1>) are selected. Additionally, AM<0> is selected by enabling the INT<0>. Then, the switches RINT and ISM1 are enabled. If the SRAM is storing “0”, the accumulation of Δ*Q_π_* to the AM is required, because *Q_TOT_* has not reached *Q_REF_* yet. In this case, *V_PIXA_* (= *V_RST_* − (*Q_π_* + *BG*)/*C_FD_*) is sampled on *C*_1_ for the accumulation. Simultaneously, the *C*_2_ for AM<0> is reset by unity-gain feedback. If the SRAM is storing “0”, no accumulation of Δ*Q_π_* is required, because *Q_TOT_* already reached *Q_REF_* in the previous *T_SUB_*, and the final Δ*Q_π_* is already stored in the AM. In this case, the reference voltage *V_REF_* is sampled on *C*_1_ instead of sampling the *V_PIXA_*. By fixing the input as a constant *V_REF_*, no accumulation is performed in the accumulator. Moreover, the AM is not reset, for preserving the stored Δ*Q_π_*. At *t*_5_, ISM2 is enabled for the accumulation. Simultaneously, *V_PIXB_* (= *V_RST_* − (*Q*_0_ + *BG*)/*C_FD_*) is input to the capacitor *C*_1_. This accumulation is performed only when the SRAM is storing “1”. Finally, the output of the CA is

(7) $$V_{o}=V_{REF}+\frac{C_{1}}{C_{2}}\frac{\left(Q_{0}-Q_{\pi }\right)}{C_{FD}}=V_{REF}+\frac{C_{1}}{C_{2}}\frac{\Delta Q_{\pi }}{C_{FD}}.$$

This operation is repeated during 128 sub-integration times. In this manner, Δ*Q_π_* is stored in one AM (AM<0>) out of the two AMs in the first frame. The other AM (AM<1>) is used for storing the next frame signals. After 128·*T_SUB_*, both the stored *A*·Δ*Q_π_*_(1)_ in AM<0> and *B*·Δ*Q_π_*_(2)_ in AM<1> are read out through the unity-gain buffer, which is used only for testing purposes, such that the second comparison of the values of |*A*·Δ*Q_π_*_(1)_ − *B*·Δ*Q_π_*_(2)_| > Δ*Q_TH_* can be performed in the external logic circuit. Even though we used the buffer circuit to read Δ*Q_π_* and performed the second comparison off-chip for the purpose of characterization, the second comparison can be easily performed using the existing comparator of the single-slope ADC. It is noteworthy that the binary quantization in the second comparison is mainly for simple post processing, e.g., optic-flow estimation [6] or motion-triggered awakening [1] that use binary information of the frame difference. In the case that analog frame difference is required to generate accurate 3D motion vectors, *A*·Δ*Q_π_*_(1)_ − *B*·Δ*Q_π_*_(2)_ can be simply generated through an additional amplifier circuit that is similar as the one used in the column amplifier.

## 5. Experimental Results

**Figure 7:**
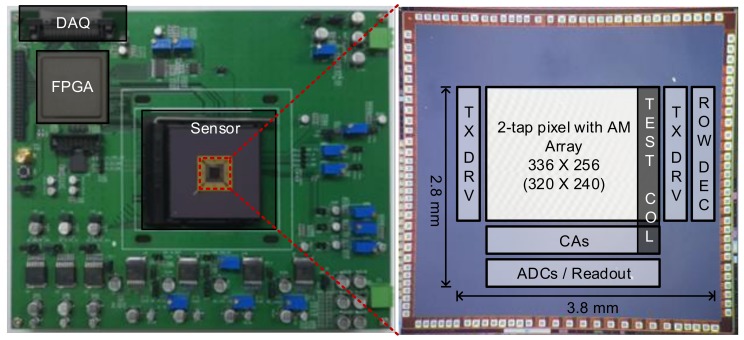
Chip characteristics and chip photograph.

A prototype chip was fabricated using a 90-nm BSI CIS process. The core size was 3.8 × 2.8 mm^2^. The light source, which was composed of IR light-emitting diodes (LEDs), was modulated at 10 MHz with a power of 40 mW for each LED. This prototype chip was originally implemented to have a split pixel array for characterizing various PPDs and pixel layouts. The pixel split was performed in a row-by-row manner. To characterize the proposed two-step comparison scheme, we implemented the accumulator with comparison logic circuits only in one column, as shown in Figure 7. The output of the accumulator was read through the unity-gain buffer. 

**Figure 8:**
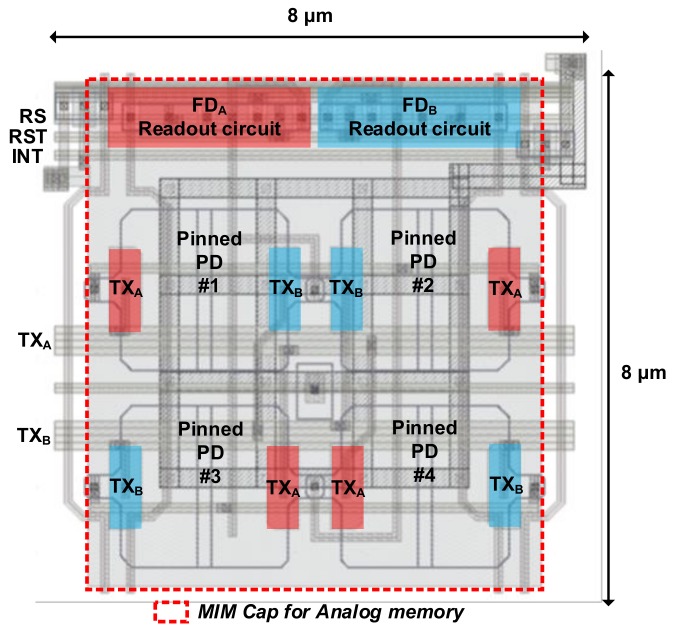
Pixel layout with the over-pixel analog memory (AM).

Table 1 presents the chip characteristics. Figure 8 shows the pixel layout with an AM. To minimize the distance of charge transfer in the PPD within a short modulation period, the size of the PPD should be small enough while guaranteeing high sensitivity. Therefore, four small PPDs were shared to provide higher sensitivity [15]. The size of each PPD was 2.3 × 2.3 μm^2^. Because the AM was implemented with an MIM capacitor using the BSI CIS process, the AM on the front side did not degrade the sensitivity. In the proposed two-step comparison scheme, the AM must be accessed 128 times using a column amplifier. However, the column amplifier was designed with a low bias current of 2 μA for power-efficient operation. The average power consumption in a column was measured as 4 μW at 20 fps, which is even smaller than the power consumption in the column-parallel ADC of conventional image sensors [16,17,18].

As illustrated in Figure 6b, the AM operates as a feedback capacitor when Δ*Q_π_* is read out through the CA. Because of the gain amplification of 8 in the CA, the capacitance variation of the AM (C_1_) affects gain term (C_1_/C_2_) in Equation (7) and induces gain fixed-pattern noise (FPN) in a column. Because the gain error from the gain FPN provides an error in the second comparison that compares the amplified Δ*Q_π_*, the result of the second comparison becomes erroneous. In order to suppress the gain FPN, the AM should be designed to have enough size such that mismatch between rows (and also between columns) are suppressed. The size of the MIM capacitor was designed to be 6.2 × 7 μm^2^. The capacitance was 278 fF. In order to prove that the gain FPN does not provide significant error if enough capacitance is used, we measured the gain FPN in the test column. The measurement result shows 0.42% FPN that corresponds to an error of the depth frame difference below 0.1 cm. 

**Figure 9:**
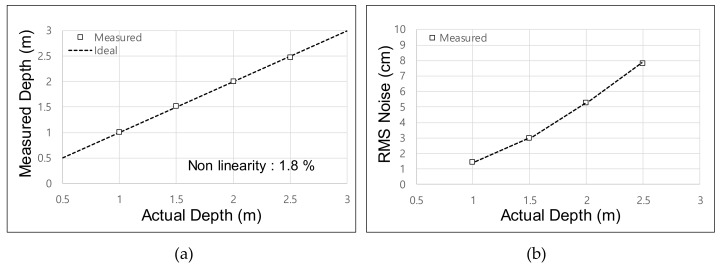
Measured depth from the fabricated sensor chip: (**a**) measured depth; (**b**) root-mean-square (RMS) noise.

Figure 9a shows the measured depth over the range of 1–2.5 m. For the four-phase operation of depth acquisition, we had to acquire Δ*Q_π_* in the first frame and Δ*Q_π_*_/2_ in the second frame. Therefore, the effective frame rate was set as 10 fps for the depth-acquisition experiment. The measured nonlinearity was 1.8%. The minimum root-mean-square (RMS) noise was measured as 1.42 cm at a distance of 1 m, as shown in Figure 9b. The frame rate of 10 fps was used only for the depth acquisition using the four-phase operation. The depth frame difference using the proposed two-step comparison scheme was measured at 20 fps because only a single frame of integration was needed to acquire Δ*Q*. We allocated a modulation time of 25 ms for all 128 sub-integration times. Reducing the modulation time enhances the frame rate but degrades the depth accuracy. The frame rate is expected to be improved by optimizing the responsivity of the PPD in further research.

**Figure 10:**
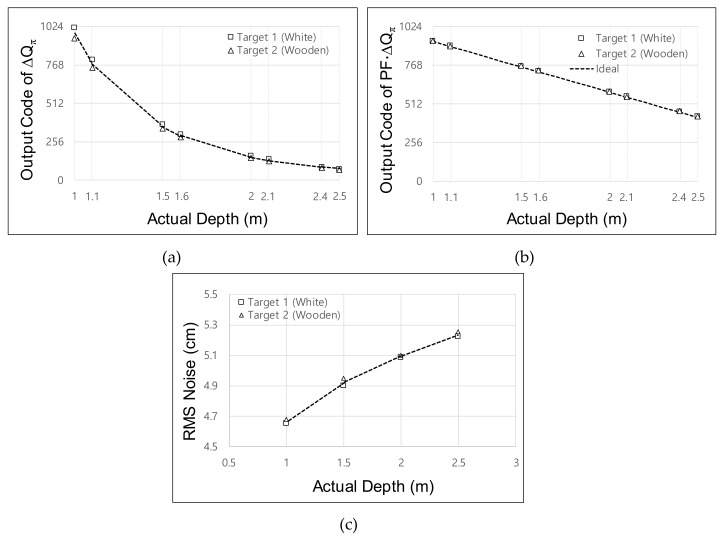
Measured Δ*Q_π_* scaled by the maximum Δ*Q_TOT_* and the results for the depth frame difference: (**a**) Δ*Q_π_* without application of a two-step comparison scheme; (**b**) Δ*Q_π_* with application of a two-step comparison scheme; (**c**) RMS error of Δ*Q_π_*.

Figure 10 shows the measured Δ*Q_π_* for acquisition of the depth frame difference. To prove that the depth frame difference can be reliably acquired regardless of the reflectivity, we measured two target objects with different reflectivities. As shown in Figure 10a, Δ*Q_π_* had a nonlinear response without the application of the two-step comparison scheme. This measured curve is similar to the curve illustrated in Figure 3a. Therefore, the accurate depth frame difference could not be detected, because even a small depth difference provided an abrupt change of Δ*Q_π_* at a short distance, whereas a large depth difference was needed to provide a sufficient change of Δ*Q_π_* at a long distance. Moreover, differences in reflectivity induced the variation of Δ*Q_π_*. Figure 10b shows the Δ*Q_π_* measured using the two-step comparison scheme. With this scheme, the output PF·Δ*Q_π_* exhibited a linear response regardless of the reflectivity. The maximum relative error between the ideal PF·Δ*Q_π_* and the measured PF·Δ*Q_π_* was 1.5% at a distance of 2.5 m. Figure 10c shows the RMS noise of the Δ*Q_π_*. Considering that the RMS error of Δ*Q_π_* can be increased by up to 5.25 cm at a 2.5-m distance, the RMS error of the depth frame difference Δ*Q_π_*_(1)_ − *ΔQ_π_*_(2)_ was <10 cm (= $\sqrt{5.25^{2}+5.25^{2}}$). Thus, the targeted resolution of the depth frame difference was 7.4 cm. In the experiment, the location of the object was adjusted in increments of 10 cm from a distance of 1 m to 2.5 m. It is noteworthy that the RMS error of Δ*Q_π_* is quite constant over the whole range of distances, whereas the RMS error of the depth measured using the conventional four-phase modulation increases along with the distance. This is because the charge is integrated up to the *Q_REF_* in the two-step comparison scheme, where *Q_REF_* cannot be set as a high value considering the maximum range of the distance. Therefore, the two-step comparison scheme provides more error in the short range, whereas it provides a similar error in the long range compared with the four-phase modulation scheme. Even though the two-step comparison scheme using the single reference *Q_REF_* provides constant error under 7.4 cm in the prototype sensor, we expect that the error can be further suppressed by using dual references, i.e., using high *Q*_REF1_ for short range and low *Q*_REF2_ for long range such that a small error can be achieved in the short range. This dual reference can be implemented spatially (implemented in dual pixels) or temporally (implemented in dual frames).

**Figure 11:**
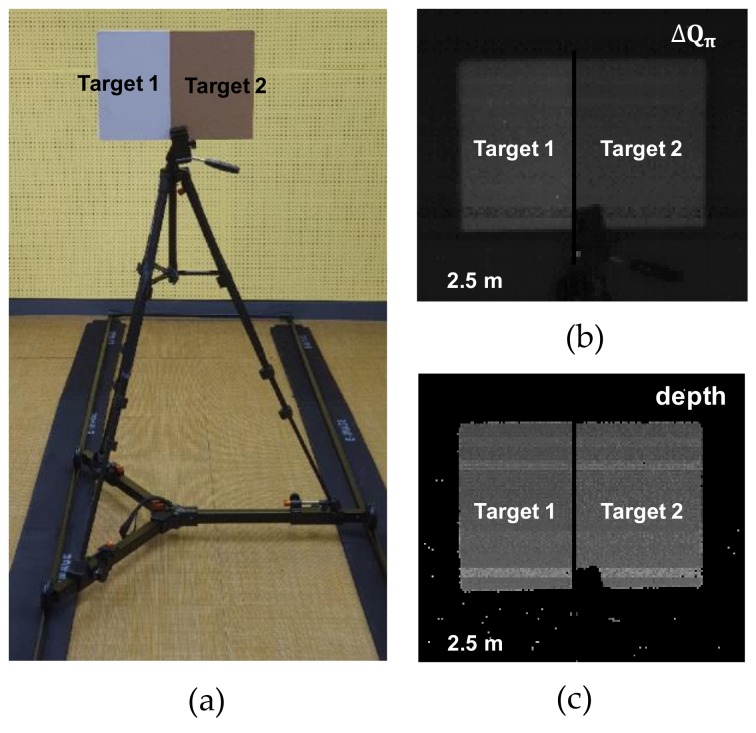
Testing environment and captured images from the fabricated sensor: (**a**) testing environment; (**b**) infrared (IR) image of Δ*Q_π_* without application of a two-step comparison scheme; (**c**) depth image with application of a conventional four-phase modulation scheme.

Figure 11 shows the testing environment and captured images from the fabricated sensor. As shown in Figure 11a, we used the hardboard with different reflectivities as a target object. Figure 11b shows the IR image of Δ*Q_π_* without the two-step comparison scheme. As illustrated in Figure 10a, output values are different owing to the reflectivity. The depth image using conventional four-phase modulation scheme is also shown in Figure 11c. No differences between reflectivities were measured, as expected. Note that the images have row patterns because the pixel split with slightly different layout was performed in a row-by-row manner for characterization purposes. 

**Figure 12:**
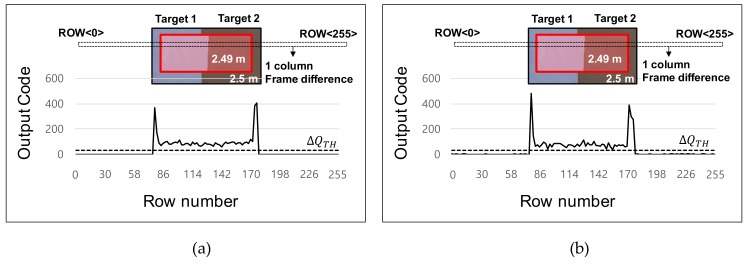

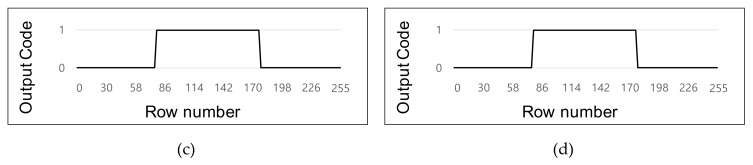
Captured line images of the depth frame difference from the test column: (**a**) line image with application of a four-phase modulation scheme; (**b**) line image with application of a two-step comparison scheme; (**c**) binary detection result with application of a four-phase modulation scheme; (**d**) binary detection result with application of a two-step comparison scheme.

Figure 12a,b show the line images of the depth frame difference that were generated from the test column with application of the four-phase modulation scheme and the proposed two-step comparison scheme, respectively. Figure 12c,d show the result of binary quantization. The threshold of the binary detection was set as 10 cm. In both results, no detection error was found regardless of the reflectivity.

**Figure 13:**
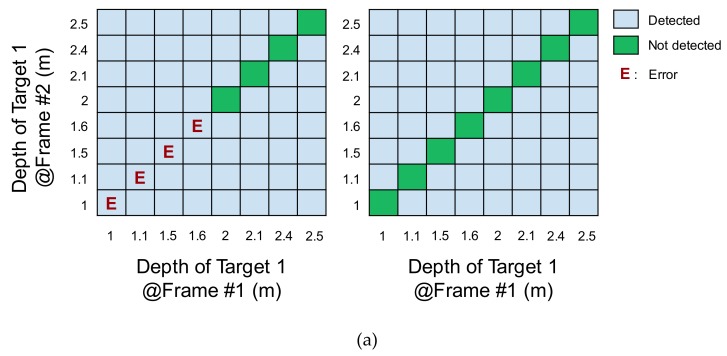

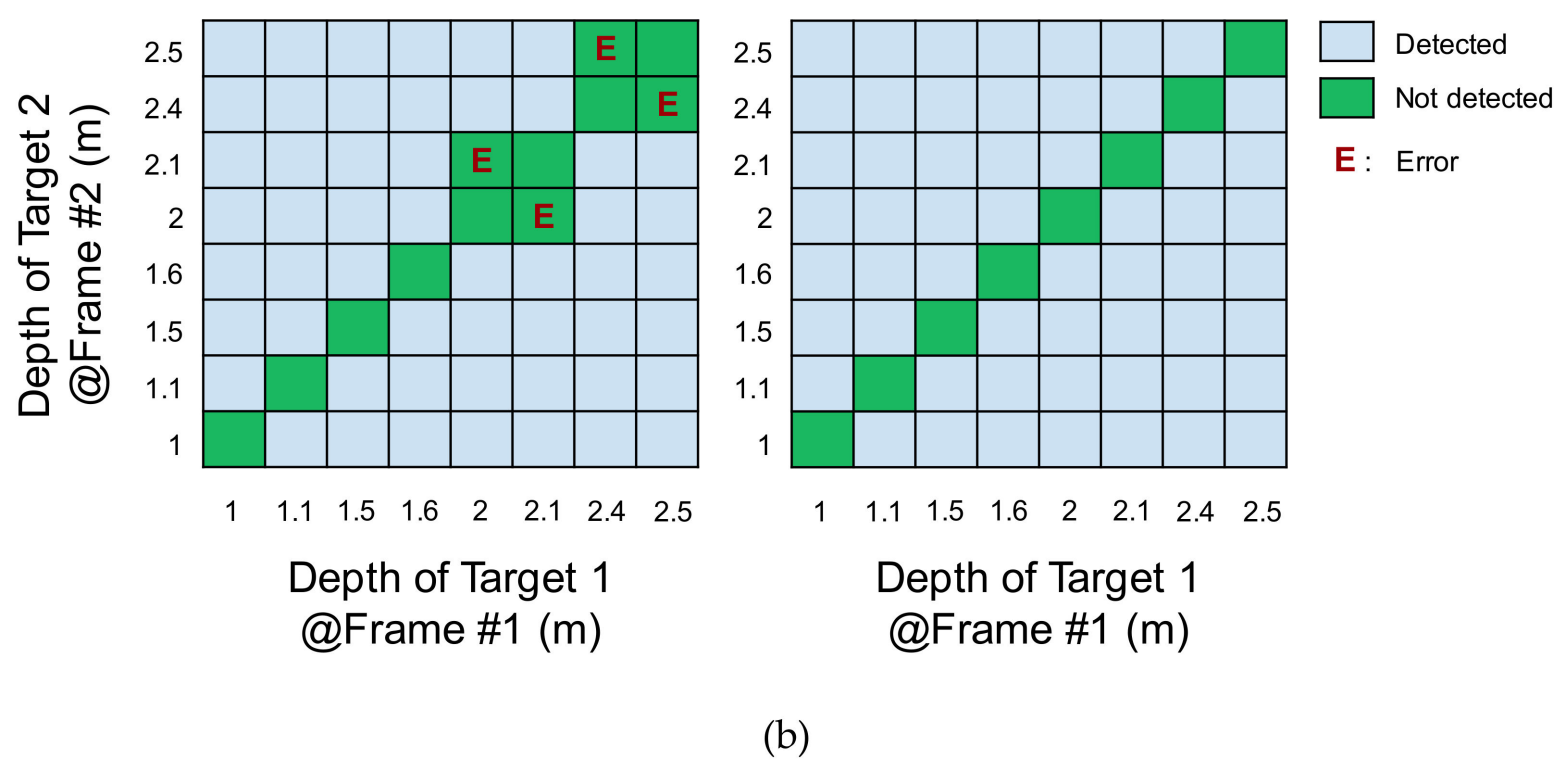
Comparison of depth frame difference results without two-step comparison (left) and with proposed circuit (right): (**a**) difference depth with same targets (Frame #1: Target 1(2), Frame #2: Target 1(2)); (**b**) difference depth with different targets (Frame #1: Target 1(2), Frame #2: Target 2(1)).

Figure 13 summarizes the result for the depth frame difference. Without the two-step comparison scheme, the frame difference was not detected in a significant portion of the range. Moreover, the detection results exhibited differences due to the different reflectivities. With the two-step comparison scheme, the frame difference was successfully detected in the entire range for both target objects with different reflectivities. 

Table 2 shows a comparison of conventional depth sensors with a four-phase modulation scheme. Regarding the performance of the depth sensor itself, our prototype sensor includes non-optimized pixels in terms of the demodulation contrast, modulation frequency, and so on. However, with a given pixel, the proposed two-step comparison scheme offers three advantages to generate on-chip depth frame difference compared with the conventional four-phase modulation scheme. Firstly, the frame rate can be doubled. In order to calculate the depth (D) using a four-phase modulation scheme, we have to acquire four signals *Q*_0_, *Q_π_*, *Q*_π/2_, and *Q*_3π/2_ in two frames. For calculating the depth frame difference, four frames of images are required. Therefore, the frame rate is reduced by half compared with the proposed two-step comparison scheme. This is disadvantageous because of motion blur for detecting moving objects. Secondly, memory requirement is reduced by half. In each frame of the depth acquisition with the four-phase modulation, two delta charges (Δ*Q_π_* and Δ*Q*_π/2_) should be stored in the frame memory in order to calculate the depth. Therefore, we need two 10-bit frame memories per pixel to store Δ*Q*_0_ and Δ*Q*_π/2_. The two-step comparison scheme reduces the requirement into a single 10-bit frame memory that stores only Δ*Q_π_*. Moreover, we used the over-pixel MIM capacitor as a frame memory without any area overhead. Thirdly, power consumption can be significantly saved. For the four-phase modulation, both the light source (LD) and pixels should be modulated with high frequency over 10 MHz in two frames. This modulation power that occurs in the two-frame modulation can be saved by the single-frame modulation of the two-step comparison scheme. In summary, the proposed depth sensor can provide both power and area efficiency while providing sufficient resolution of the depth frame difference; thus, the sensor is applicable to gesture sensors, object trackers, motion-triggered surveillance, vacuum robot navigators, etc.

## 6. Conclusions

An iTOF depth sensor with integrated circuits that detects the depth frame difference was proposed. To detect the accurate difference of the depth in successive frames regardless of the reflectivity, we proposed a two-step comparison scheme with an amplifier-based accumulator and an over-pixel AM. To suppress the error arising from the nonlinear response of light-dependent charges, we used adaptive modulation times and 128 sub-integration times. According to experimental results, a 10-cm depth frame difference was successfully detected at a 2.5-m distance with 3% relative error according to the difference in the reflectivity. Owing to the single-frame operation, the measured power consumption was 10.7 μW for each column, and the power consumption of the modulation driver circuits was 6.7 μW for each column. Additionally, compact implementation of <3.8 × 2.8 mm^2^ was possible without external frame memories. Therefore, the proposed iTOF sensor can be utilized in a variety of applications, including surveillance, gesture recognition, object tracking, and navigation.

## Figures and Tables

**Table 1:** Chip characteristics.

Parameter	Value
Process	90-nm BSI CMOS image sensor
Core size	3.8 × 2.8 mm^2^
Pixel size	8 × 8 μm^2^ (fill factor: 39%)
Frame rate	20 fps
Powers supply voltage	2.8 V (pixel), 1.8 V (analog), 1.2 V (digital)
Power consumption/ frame (per column)	4 μW (at 128 sub-integration times)
Power [μW]	8.8
Range	1–2.5 m
(De)modulation frequency	10 MHz
Light source	850-nm IR LED
Demodulation contrast	74%

**Table 2:** Comparison of conventional depth sensors with a four-phase modulation scheme.

Parameter	[19], JSSC 2018	[20], TCAS I 2019	[13], JSSC 2014	This Work	
				4 Phase Operation	2 Step Comparison Operation
Process	90 nm	350 nm	110 nm	90 nm	
Demodulation Frequency	100 MHz	5 MHz	12.5 MHz	10 MHz	
Demodulation contrast	85%	64%	50%	74%	
Pixel	10 × 10 μm^2^	48 × 48 μm^2^	5.9 × 5.9 μm^2^	8 × 8 μm^2^	
Fill factor	>80% (w/microlens)	17.40%	24%	39%	
Range	-	0.5 m ~ 2 m	0.75 m ~ 4 m	1 m ~ 2.5 m	
Integrated depth frame difference	×	×	×	×	○
Resolution	320 × 240	24 × 24	84 × 64	336 × 256	1 × 128
Frame-rate	-	100	10	10	20
External memory	-	-	-	○	×
Image Signal Processor	-	-	-	○	×
Illumination power	-	500 mW	650 μW/cm^2^ @ 1m	240 mW	120 mW
